# The Actinomyosin Motor Drives Malaria Parasite Red Blood Cell Invasion but Not Egress

**DOI:** 10.1128/mBio.00905-18

**Published:** 2018-07-03

**Authors:** Abigail J. Perrin, Christine R. Collins, Matthew R. G. Russell, Lucy M. Collinson, David A. Baker, Michael J. Blackman

**Affiliations:** a Malaria Biochemistry Laboratory, The Francis Crick Institute, London, United Kingdom; b Electron Microscopy Science Technology Platform, The Francis Crick Institute, London, United Kingdom; c Faculty of Infectious and Tropical Diseases, London School of Hygiene & Tropical Medicine, London, United Kingdom; Washington University School of Medicine

**Keywords:** GAP45, Plasmodium falciparum, egress, glideosome, invasion, malaria

## Abstract

Apicomplexa are obligate intracellular parasites that actively invade, replicate within, and egress from host cells. The parasite actinomyosin-based molecular motor complex (often referred to as the glideosome) is considered an important mediator of parasite motility and virulence. Mature intracellular parasites often become motile just prior to egress from their host cells, and in some genera, this motility is important for successful egress as well as for subsequent invasion of new host cells. To determine whether actinomyosin-based motility is important in the red blood cell egress and invasion activities of the malaria parasite, we have used a conditional genetic approach to delete *GAP45*, a primary component of the glideosome, in asexual blood stages of Plasmodium falciparum. Our results confirm the essential nature of GAP45 for invasion but show that P. falciparum does not require a functional motor complex to undergo egress from the red blood cell. Malarial egress therefore differs fundamentally from induced egress in the related apicomplexan Toxoplasma gondii.

## IMPORTANCE

Clinical malaria results from cycles of replication of single-celled parasites of the genus *Plasmodium* in red blood cells. Intracellular parasite replication is followed by a highly regulated, protease-dependent process called egress, in which rupture of the bounding membranes allows explosive release of daughter merozoites which rapidly invade fresh red cells. A parasite actinomyosin-based molecular motor (the glideosome) has been proposed to provide the mechanical force to drive invasion. Studies of the related parasite Toxoplasma gondii have shown that induced egress requires parasite motility, mediated by a functional glideosome. However, whether the glideosome has a similar essential role in egress of malaria merozoites from red blood cells is unknown. Here, we show that although a functional glideosome is required for red blood cell invasion by Plasmodium falciparum merozoites, it is not required for egress. These findings place further emphasis on the key role of the protease cascade in malarial egress.

## INTRODUCTION

Clinical malaria results from the proliferation of *Plasmodium* parasites within host red blood cells (RBCs). Specialized developmental forms known as merozoites invade RBCs, within which they multiply asexually, forming a mature schizont that undergoes a segmentation or budding process to produce daughter merozoites. These are released into the bloodstream in a process termed egress. Within seconds to minutes of egress, the free merozoites bind to and actively invade new RBCs, amplifying the infection and eventually leading to disease.

Like most invasive developmental forms (zoites) of apicomplexan parasites, the merozoite surface has a pellicular architecture comprising a plasma membrane overlying a closely apposed set of flattened vesicular sacs which together form a double-membrane structure known as the inner membrane complex (IMC). The ~20- to 40-nm space between the plasma membrane and the IMC (1, 2) contains a set of interacting proteins often referred to as the glideosome (3), which collectively constitute an unusual actinomyosin-based contractile system. While there is some controversy over the precise topology of the glideosome (4), key conserved constituents include short actin filaments, a class XIV myosin (MyoA), associated myosin light chains (called ELC and MTIP in *Plasmodium*), a polytopic IMC protein called gliding-associated protein 40 (GAP40), an integral membrane protein called GAP50, and an additional protein called GAP45 (2, 5–7). The latter is a 45-kDa myristoylated and palmitoylated protein encoded by a single exon, expressed in mature schizonts, and trafficked to the IMC of developing *Plasmodium* merozoites (8, 9). GAP45, which is predicted to include a central coiled-coil segment followed by a highly conserved C-terminal region of unknown secondary structure (5), is thought to bridge the plasma membrane and IMC by being anchored at its C terminus within the IMC and attached to the plasma membrane through acyl moieties near its N terminus. Importantly, GAP45 has the additional function of recruiting both MyoA and MTIP to the IMC through interactions with the conserved C-terminal region (2, 5).

The glideosome is largely conserved between apicomplexan genera, and there is good evidence in both *Plasmodium* and the related apicomplexan Toxoplasma gondii that it plays essential roles in generating the force that drives zoite motility (2, 10–13). Multiple lines of evidence indicate that glideosome function is also important for all known host cell invasion processes mediated by apicomplexan parasites (reviewed recently by Frénal et al. [14]). In the case of the *Plasmodium* species responsible for the majority of malaria fatalities, Plasmodium falciparum, invasion of RBCs by merozoites is initiated through reversible receptor-ligand interactions between surface proteins of the parasite and target cell (reviewed in reference 15). This is quickly followed by transient deformation of the RBC membrane and reorientation of the merozoite such that its apical pole contacts the RBC surface. A zone of apparent membrane thickening then appears between the apical prominence of the merozoite and the host RBC membrane, corresponding to a high-affinity interaction known as the tight junction. The merozoite then propels itself into the host cell using its actinomyosin motor; to do this, the glideosome complex engages with transmembrane adhesin molecules which are themselves bound to receptors on the RBC surface. Rearward translocation of the adhesins effectively drags the RBC membrane around the merozoite, forming a nascent parasitophorous vacuole (PV) into which the parasite enters. The PV then seals and pinches off from the RBC membrane, forming a discrete intracellular compartment within which the parasite initiates its replicative growth phase (6, 16, 17). Successful invasion is generally followed by a short period of host RBC echinocytosis, thought to result from transient dysregulation of RBC membrane phospholipid distribution or cytoskeletal integrity, mediated by calcium flux or discharge of merozoite secretory organelles called rhoptries (17, 18).

Detailed studies of asexual blood forms of P. falciparum have shown that egress is also a multistep process in which permeabilization and then breakdown of the PV membrane (PVM) are rapidly followed by permeabilization and then rupture of the host RBC membrane (19–23). Egress is regulated by the single parasite cGMP-dependent protein kinase (PKG), which, in cooperation with a calcium-dependent protein kinase called CDPK5 (24, 25), triggers a proteolytic cascade initiated by the discharge into the PV lumen of a parasite subtilisin-like serine protease called SUB1 (26, 27). SUB1 is required for PVM rupture and also activates members of the SERA family of papain-like PV proteins, including a putative cysteine protease called SERA6 which is required for RBC membrane rupture (22, 28). SUB1 additionally cleaves a range of other substrates, including the major merozoite surface protein MSP1, which appears to interact with the RBC cytoskeleton to promote egress (29, 30).

PKG- and protease-dependent processes have also been implicated in egress of sporozoites from oocysts and gametes from RBCs in the mosquito stages of the *Plasmodium* life cycle (31, 32), as well as egress of liver-stage merozoites (33), and PKG is also required for T. gondii egress (34–36). However, in addition to these strategies, active zoite motility has been implicated in facilitating egress. Activation of *Plasmodium* sporozoite motility appears to be a prerequisite for egress from oocysts (37), and in T. gondii, egress can be induced by treatment of parasite-infected cells with calcium or potassium ionophores, which induce rapid onset of parasite motility and subsequent egress (38, 39). This ionophore-induced egress in T. gondii, which has been widely used to study the molecular mechanisms underlying egress, is dependent on the presence of the glideosome components TgMLC1 (the orthologue of MTIP; Tg stands for *T. gondii*) and TgGAP45 (2, 10, 37); thus, deletion of the central glideosome component *TgGAP45* severely impairs the ability of T. gondii to undergo both ionophore-induced egress and invasion (2, 10). In T. gondii, phosphorylation of MyoA is important for its function in motility and induced egress (40, 41), while in P. falciparum asexual blood stages, a phosphoproteomic analysis has shown that the glideosome components MyoA, GAP40, and GAP45 are also phosphorylated in a PKG-dependent manner in mature schizonts (42). This suggests that the merozoite glideosome may be primed or activated by phosphorylation just prior to egress. In support of this concept, several reports have noted that immediately following PVM rupture but just prior to final egress, the still-intracellular P. falciparum merozoites display increased motility (e.g., references 21 and 22). Whether this movement reflects glideosome activation that is important in egress is unknown. While free P. falciparum merozoites have not been observed actively gliding on solid substrates, they clearly exert pulling forces on erythrocytes upon contact even under circumstances where this does not lead to productive invasion (17, 43), and merozoites of the closely related apicomplexan *Babesia* do glide (44). Collectively, the accumulated evidence strongly implicates actinomyosin-based motility in RBC invasion. However, whether a functional glideosome complex is required for egress of blood-stage *Plasmodium* merozoites has not been addressed.

Here, we have used conditional mutagenesis to examine the function of the P. falciparum glideosome in RBC egress and invasion. Our results conclusively demonstrate that glideosome function is dispensable for egress.

## RESULTS

### Conditional disruption of the P. falciparum *GAP45* gene shows that it is essential for parasite viability.

**FIG 1:**
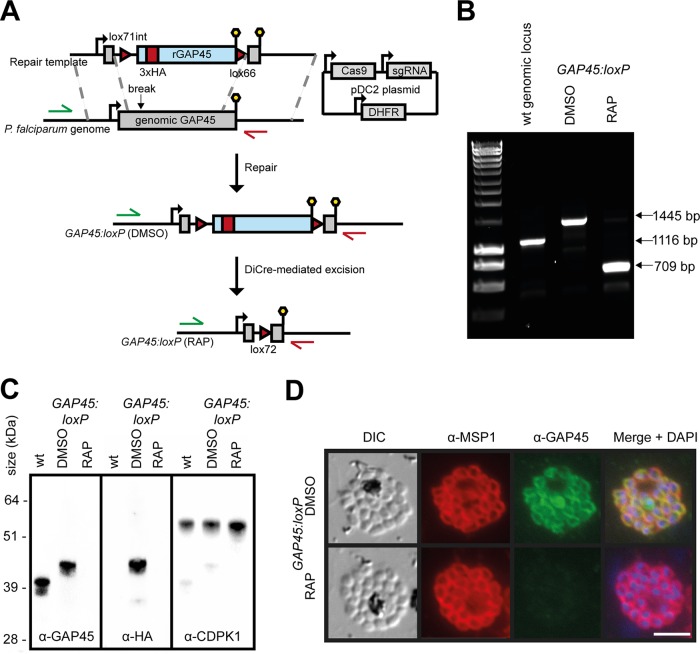
Efficient conditional silencing of *GAP45* expression. (A) Schematic representation of the Cas9-based approach for conditional disruption of the *GAP45* gene in the DiCre-expressing P. falciparum line B11 (a derivative of 1G5DC; see Materials and Methods). Green and red arrows represent the positions of hybridization of oligonucleotide primers used in panel B. Lollipops, translational stop codons. Red arrowheads, *loxP* sites. Light blue boxes, recodonized sequence. Red block, introduced HA3 epitope tag. (B) Diagnostic PCR confirming successful modification of the *GAP45* locus in a *GAP45:loxP* P. falciparum clone and RAP-mediated excision of the floxed *GAP45-HA3* transgene. Mock-treated parasites were treated with vehicle only (DMSO). (C) Western blots showing successful epitope tagging and rapamycin-inducible ablation of GAP45-HA3 expression. Note that HA3-tagged GAP45 migrates more slowly on SDS-PAGE gels than unmodified GAP45 due to its increased molecular mass. Expression of the unrelated parasite kinase CDPK1 was used as a loading control. (D) IFA showing the subcellular localization of GAP45-HA3 to the periphery of intracellular merozoites in mock-treated *GAP45:loxP* parasites and the loss of GAP45-HA3 upon RAP treatment. Over 99% of schizonts examined by IFA were GAP45 and HA negative following RAP treatment. Bar, 5 µm.

We used Cas9-enhanced, marker-free targeted homologous recombination to modify the *GAP45* locus (PlasmoDB PF3D7_1222700) in an improved (see Materials and Methods) P. falciparum line stably expressing DiCre (45). To do this, we replaced the endogenous *GAP45* open reading frame (ORF) with a synthetic recodonized sequence modified by the inclusion of a triple-hemagglutinin (HA3) epitope tag within an internal low-complexity region of the ORF; previous work has shown that incorporation of a tag at this position is tolerated by the parasite and so presumably does not interfere with GAP45 function (46). The recodonized gene included close to its 5′ end a *SERA2* intron containing a *lox71* site and was followed by a *lox66* site downstream of the translational stop site at its 3′ end (Fig. 1A). Clonal lines of the modified parasites (called *GAP45:loxP*) were generated by limiting dilution, and the structure of the modified *GAP45* locus in these clones was verified by PCR (Fig. 1B) and nucleotide sequencing. Examination of mature segmented *GAP45:loxP* schizonts by Western blotting and immunofluorescence assay (IFA) using anti-HA or anti-GAP45 antibodies confirmed expression of the tagged transgene and showed that the GAP45-HA3 protein localized as expected to the periphery of intracellular merozoites (Fig. 1C and D). Rapamycin (RAP) treatment of synchronized *GAP45:loxP* parasites to induce DiCre activity resulted in the anticipated excision of the *GAP45-HA3* gene (Fig. 1B) and completely abolished the Western blotting and IFA signals by the end of the erythrocytic cycle in which the parasites were RAP treated (cycle 0) (Fig. 1C and D). This indicated rapid and efficient silencing of GAP45 expression within the course of a single erythrocytic cycle (~48 h). The ΔGAP45 schizonts developed at the same rate as mock-treated *GAP45:loxP* parasites and appeared morphologically normal at the end of cycle 0 (Fig. 1D), suggesting that GAP45 is not essential for schizont development.

**FIG 2:**
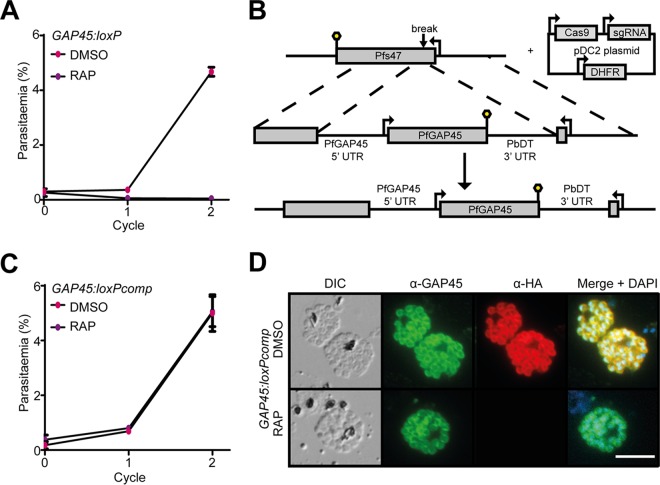
GAP45 is essential for proliferation of asexual blood-stage P. falciparum. (A) Growth curves showing replication of the *GAP45-HA3:loxP* parasite line following RAP or mock (DMSO) treatment. RAP-induced excision of the *GAP45-HA3* locus produced parasites that were unable to replicate *in vitro*. Means from three replicates plotted. Error bars, standard deviations. (B) Schematic representation of the genetic complementation strategy used to introduce a second copy of the *GAP45* gene and its promoter sequence into the *Pfs47* locus of a *GAP45-HA3:loxP* parasite clone, generating the *GAP45-HA3:loxPcomp* line. Lollipops, translational stop codons. (C) Growth curves showing proliferation of the *GAP45-HA3:loxPcomp* parasite line following treatment with RAP or DMSO. The presence of the second *GAP45* gene copy at the *Pfs47* locus allowed the parasites to grow normally following RAP-mediated excision of the floxed *GAP45-HA3* gene at the endogenous *GAP45* locus. Means from three replicates plotted. Error bars, SD. (D) IFA showing continued expression of GAP45 from the modified *Pfs47* locus following RAP-mediated silencing of GAP45-HA3 expression in *GAP45-HA3:loxPcomp* parasites. Bar, 5 µm.

To determine the effects of GAP45 loss on longer-term parasite viability, growth of RAP- and mock (dimethyl sulfoxide [DMSO])-treated *GAP45:loxP* parasites was monitored in parallel over the ensuing cycles. As shown in Fig. 2A, the RAP-treated *GAP45:loxP* parasites did not proliferate in culture, indicating a significant growth defect. To assess whether this was due solely to loss of GAP45 expression rather than exposure to RAP or any other consequence of DiCre induction, a *GAP45:loxP* parasite clone was further modified by integrating a second (untagged) copy of the *GAP45* gene, under the control of its native promoter sequence, into the genomic *Pfs47* locus (Fig. 2B). Treatment with RAP of the resulting parasite clone (called *GAP45:loxPcomp*) ablated expression of the HA-tagged floxed GAP45 gene as expected but did not ablate expression of GAP45 from the second gene copy (Fig. 2C and D). Importantly, these RAP-treated *GAP45:loxPcomp* parasites continued to replicate at the same rate as mock-treated parasites (Fig. 2C), indicating efficient genetic complementation of the mutant. Collectively, these results convincingly demonstrated that GAP45 is indispensable for long-term survival of asexual blood-stage P. falciparum.

### ΔGAP45 parasites fail to assemble an intact glideosome but show no discernible defect in IMC formation.

**FIG 3:**
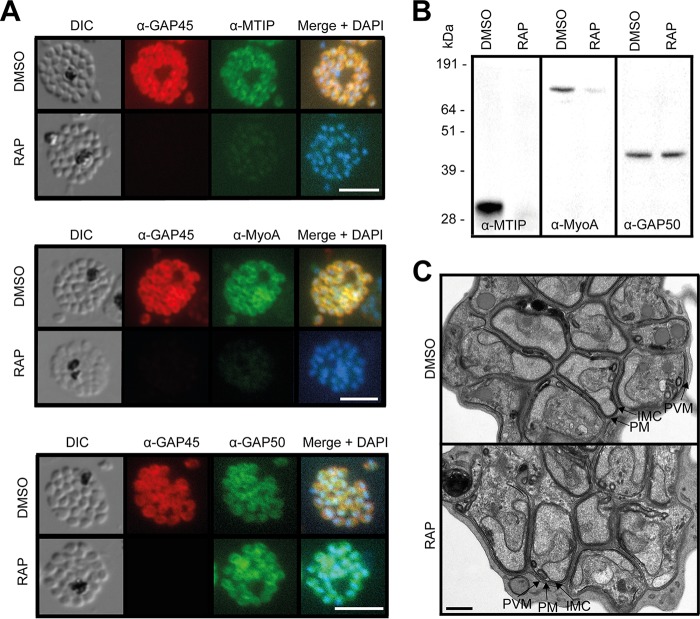
ΔGAP45 parasites show defects in expression of the glideosome components MyoA and MTIP. (A) IFA showing the subcellular localization of GAP45-HA3, MTIP, MyoA, and GAP50 in segmented schizonts of ΔGAP45 (RAP) and mock-treated (DMSO) *GAP45:loxP* parasites. Loss of GAP45-HA3 also resulted in loss of detection of MTIP and MyoA at the IMC upon RAP treatment. Bars, 5 µm. (B) Western blots showing the decreased overall abundance of MTIP and MyoA proteins in the absence of GAP45. (C) TEM images showing similar merozoite and IMC morphologies in GAP45-HA3 and ΔGAP45 parasites. Merozoite plasma membrane (PM), merozoite IMC, and schizont PVM are indicated. Bar, 0.5 µm.

Extensive dissection of glideosome assembly in T. gondii has resulted in a model in which the C-terminal domain of GAP45 recruits the TgMLC1/TgMyoA complex and binds to the IMC via GAP50 or GAP40 (2). We therefore used IFA and Western blotting to examine whether MTIP and MyoA were expressed and correctly recruited to the IMC in the ΔGAP45 parasites. As shown in Fig. 3A and B, neither MTIP nor MyoA was expressed at wild-type levels in ΔGAP45 schizonts, suggesting that the stable expression of these proteins was dependent upon the presence of GAP45. In contrast, the other major glideosome component, GAP50, was detectable at apparently wild-type levels at the periphery of intracellular ΔGAP45 merozoites. These data demonstrated a defect in motor complex assembly in the ΔGAP45 parasites and support the current model of glideosome assembly.

GAP45 has been suggested to be an important determinant of the stability and correct positioning of the inner membrane complex both in developing *Plasmodium* ookinetes (11) and during invasion by T. gondii tachyzoites (2). To examine whether loss of GAP45 resulted in defects in IMC formation in intracellular P. falciparum blood stages, we used transmission electron microscopy (TEM) to examine segmented ΔGAP45 schizonts at high resolution. This revealed no discernible impairment in IMC development or morphology in the intracellular merozoites (Fig. 3C). It was concluded that GAP45 does not play an essential role in maintaining the structural integrity of the IMC in mature intracellular P. falciparum merozoites.

### GAP45 is dispensable for egress and microneme discharge but essential for RBC invasion.

**FIG 4:**
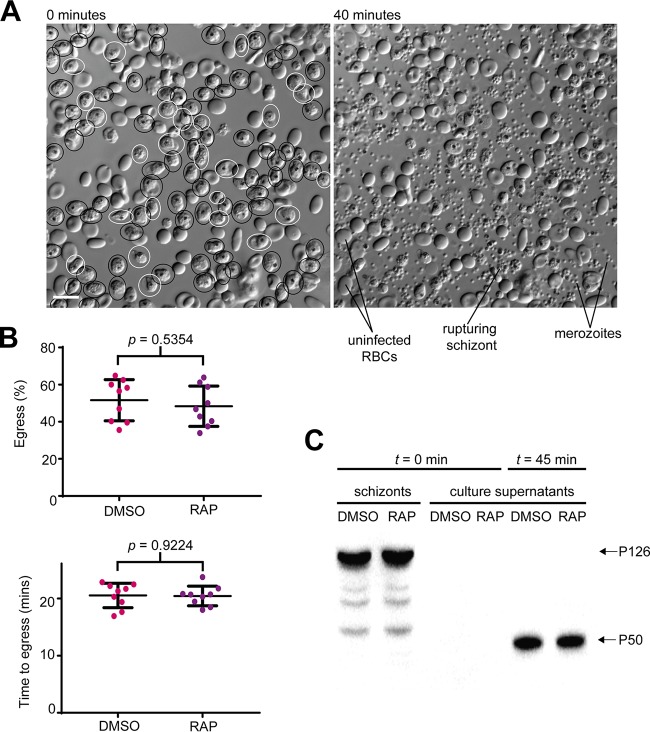
GAP45 is not required for egress. (A) First and final frames from a 40-min time-lapse video of ΔGAP45 schizonts undergoing egress. Schizonts from the first frame that subsequently rupture over the course of the video are circled in black (approximately 80% of the total population). Schizonts that do not rupture are circled in white. (B) Quantification of egress in mock-treated (DMSO) and RAP-treated (ΔGAP45) *GAP45:loxP* parasites. The top plot shows the proportion of schizonts that undergo egress in each 40-min video. The bottom plot shows the time taken for the schizonts in each video to egress. There were no significant differences between the RAP- and mock-treated populations in the efficiency or kinetics of egress. (C) Western blot showing similar levels of the P50 form of SERA5 (which results from egress-associated proteolytic processing of the P126 precursor [27, 58]) released into culture supernatants of DMSO- and RAP-treated *GAP45:loxP* schizonts following 45 min of egress.

To characterize the growth impairment displayed by ΔGAP45 parasites, we used time-lapse live video microscopy to monitor the morphology and kinetics of egress following maturation of cycle 0 schizonts. As shown in Fig. 4A and B and Movie S1 in the supplemental material, ΔGAP45 parasites underwent egress at a rate and frequency indistinguishable from those of control mock-treated counterparts. Interestingly, the ΔGAP45 merozoites also displayed the typical enhanced intracellular motility often observed immediately following PVM rupture (but prior to RBC membrane rupture), showing that this movement is not dependent on an intact glideosome. Consistent with the unmodified egress kinetics, the P50 form of the soluble PV protein SERA5, a marker of egress, was released at comparable levels into the cell culture supernatants of ΔGAP45 and control parasites (Fig. 4C). It was concluded that loss of GAP45 has no effect on parasite egress.

10.1128/mBio.00905-18.1 MOVIE S1  GAP45 is not required for egress. Time-lapse video microscopy showing egress of mock-treated parasites (unstained) and RAP-treated ΔGAP45 parasites (blue, stained with Hoechst stain). Download MOVIE S1, AVI file, 1.8 MB. Copyright © 2018 Perrin et al. 2018 Perrin et al. This content is distributed under the terms of the Creative Commons Attribution 4.0 International license.

**FIG 5:**
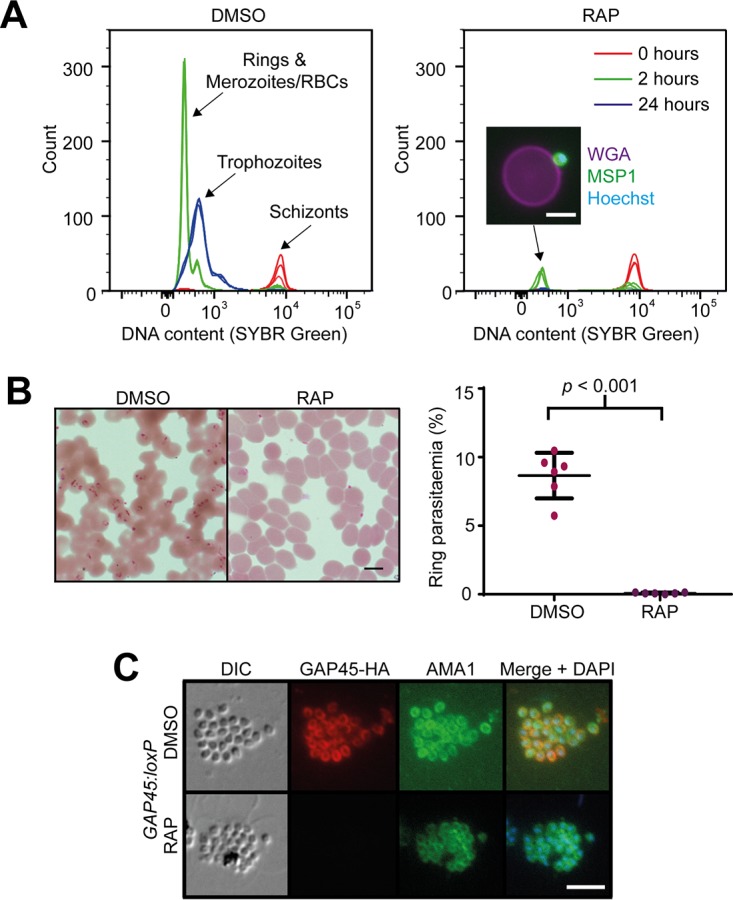
GAP45 is required for invasion but not for microneme discharge. (A) Flow cytometry-based invasion assay showing failure of ΔGAP45 parasites to develop from schizonts (*t* = 0 h) through rings (*t* = 2 h) to trophozoites (*t* = 24 h). The inset image shows a merozoite, stained with anti-MSP1 antibody and an Alexa 488 secondary antibody (green) and Hoechst stain (blue), bound to an uninfected RBC stained with wheat germ agglutinin (WGA) conjugated to Alexa 647 (magenta). Bar, 5 µm. Comparable results were observed in five additional flow cytometry-based invasion assays. (B) Giemsa-stained thin films showing the presence of ring-stage parasites in the invaded mock-treated (DMSO) *GAP45:loxP* parasites and absence of rings in the RAP-treated (ΔGAP45) *GAP45:loxP* parasites. Bar, 5 µm. This was observed in more than 10 experiments. Quantification of rings by manual counting of Giemsa-stained films in one representative experiment is shown (right). (C) IFA showing relocalization of microneme protein AMA1 to the merozoite periphery in both mock-treated and ΔGAP45 parasites. Bar, 5 µm.

Since the ΔGAP45 parasites were able to egress efficiently, we reasoned that a defect in RBC invasion was the most likely cause of the parasites’ failure to propagate. Using a flow cytometry-based assay alongside light microscopy, we found that released ΔGAP45 merozoites bind to new RBCs but fail to form the ring stages indicative of successful invasion (Fig. 5A and B). To further examine the ΔGAP45 phenotype, we again used live video microscopy to examine in real time interactions between released merozoites and host RBCs (Movies S2 and S3). This confirmed that ΔGAP45 merozoites fail to invade. Furthermore, in contrast to what is usually observed upon interactions between control merozoites and target RBCs, cells contacted by ΔGAP45 merozoites showed none of the surface deformation generally associated with successful invasion. Interestingly, however, despite the absence of invasion, some of the contacted RBCs did respond by undergoing echinocytosis (observed in eight of 21 ΔGAP45 egress events filmed [Movie S3]), a phenomenon previously noted in parasites treated with the actin polymerization inhibitor cytochalasin D (17).

10.1128/mBio.00905-18.2 MOVIE S2  Mock-treated (DMSO) merozoites deform red blood cells, invade, and induce echinocytosis. Download MOVIE S2, AVI file, 0.6 MB. Copyright © 2018 Perrin et al. 2018 Perrin et al. This content is distributed under the terms of the Creative Commons Attribution 4.0 International license.

10.1128/mBio.00905-18.3 MOVIE S3  ΔGAP45 merozoites do not deform or invade RBCs but can induce echinocytosis. Download MOVIE S3, AVI file, 0.8 MB. Copyright © 2018 Perrin et al. 2018 Perrin et al. This content is distributed under the terms of the Creative Commons Attribution 4.0 International license.

Invasion requires regulated discharge of micronemes, a set of secretory organelles found at the apical end of merozoites. In the case of the microneme protein apical membrane antigen 1 (AMA1), which plays an essential role in invasion, discharge is often detectable just prior to egress (26). IFA analysis of highly mature, segmented schizonts showed that translocation of AMA1 to the surface of ΔGAP45 parasites occurred as normal (Fig. 5C), indicating that microneme secretion is unaffected by the absence of GAP45. Collectively, we conclude that the defect in invasion in ΔGAP45 parasites is likely a consequence of a lack of actinomyosin motor activity.

## DISCUSSION

Despite their common ancestry, apicomplexan parasites display considerable diversity in morphology, host range, and target cell type. For example, P. falciparum merozoites invade only mammalian red blood cells, while T. gondii tachyzoites can invade nucleated cells of virtually any warm-blooded host (but not enucleated mammalian red blood cells). Much of our knowledge of glideosome structure and biogenesis has emanated from work in T. gondii, and as our results show, it is not unreasonable to extrapolate from some of those findings to draw conclusions about glideosome function more generally across the phylum. However, our results here also highlight some key differences in glideosome function between different apicomplexan zoites.

Our conditional gene disruption strategy allowed us to confirm that GAP45 is indeed an essential protein in P. falciparum and that it is required for glideosome assembly. The continued expression of GAP50, but not MTIP or MyoA, at the parasite periphery in the absence of GAP45 supports the current model of motor assembly studied in particular detail in *Toxoplasma*, whereby GAP45 recruits the MTIP/MyoA complex to the glideosome (2).

P. falciparum merozoites appear to form a normal IMC and pellicle in the absence of GAP45, which was notable in the light of previous studies on other apicomplexan zoites. In those studies, deletion of different domains of T. gondii GAP45 suggested that the acylation and length of the coiled-coil domain were important, leading to the suggestion that GAP45 regulates the spacing and structural stability of the IMC membranes (2). Downregulation of Plasmodium berghei GAP45 in sexual stages prevented parasites from undergoing the transition to ookinetes, and examination of these mutant zygotes showed that the IMC was detached from the plasma membrane, consistent with the role for GAP45 described in T. gondii of bridging and stabilizing the space between the IMC and the plasma membrane (11). T. gondii parasites possess GAP45 paralogues called GAP70 and GAP80, which can reportedly interact with MyoA and MyoC, respectively, to assemble glideosome structures (47). There are no obvious orthologues of GAP70 or GAP80 in *Plasmodium* species, and so it is unlikely that ΔGAP45 parasites assemble any glideosome-like structures. In spite of this, merozoite development and membrane structures appeared normal in ΔGAP45 merozoites, implying that the structural integrity of these relatively small zoites is not dependent on proteins that that span the IMC-plasma membrane space or, alternatively, that there are proteins in that compartment other than GAP45 that can perform this function.

The most important conclusion from our work is that egress of P. falciparum merozoites proceeds with normal kinetics and morphology in parasites lacking an intact glideosome, indicating that the actinomyosin-based motor is not involved in egress. This is consistent with evidence from a recent conditional knockout of parasite actin by Das and colleagues (48) and shows unambiguously that asexual blood-stage *Plasmodium* parasites rely solely on motor-independent mechanisms for egress. This is an important difference from T. gondii tachyzoites, which appear to use motility to break out of their host cell and, in the absence of key glideosome components, are severely impaired in their ability to egress (2, 10). Intriguingly, we observed that egressing ΔGAP45 merozoites still exhibited the commonly observed sudden increase in motility upon PVM rupture, which generally occurs just prior to egress. We therefore consider it reasonable to conclude that this motion is not driven by the actinomyosin motor and may be purely diffusive (i.e., Brownian) and/or driven by osmotic pressure.

We have confirmed that the actinomyosin motor complex has an essential role in RBC invasion by P. falciparum parasites, demonstrated by a complete absence of ring-stage parasites following egress of ΔGAP45 schizonts. This contrasts with recent observations in which T. gondii tachyzoites are able to invade (albeit significantly less efficiently) in the absence of key motor components (10). Host cell actin dynamics have been shown to contribute to invasion of T. gondii tachyzoites (49–51) and P. berghei sporozoites (50), which may explain the observation of glideosome-independent invasion events. Our results here may suggest that the comparatively simple architecture of the RBC does not support invasion in the absence of a canonical parasite glideosome.

Our observations of interactions between ΔGAP45 merozoites and RBCs show by using a conditional genetic method what previous studies have shown by using chemical inhibition of the parasite actinomyosin motor using cytochalasin D (17). We confirm that the characteristic deformations of the RBC surface upon interaction with a merozoite are the result of pulling forces generated by engagement of the parasite motor, since these deformations do not occur in the absence of an intact glideosome. Successful RBC invasion is often followed by an enigmatic and transient change from a smooth biconcave to a “stellate” RBC morphology, a process known as echinocytosis. Remarkably, ΔGAP45 merozoites were able to induce this change in RBC morphology while still clearly associated with the RBC surface. Since echinocytosis is thought to be dependent on rhoptry discharge (17), we conclude that the activity of the parasite motor is not required for discharge of these apical organelles.

In summary, our results conclusively demonstrate the mechanistic uncoupling of egress and invasion in asexual blood-stage P. falciparum parasites; invasion is dependent on an intact canonical actinomyosin motor, but egress occurs independently of motor function and relies instead on alternative mechanisms such as the proteolytic dismantling of the PVM and RBC membrane (28).

## MATERIALS AND METHODS

### Plasmid construction.

The endogenous *GAP45* gene in the B11 DiCre-expressing P. falciparum clone (see below) was replaced with a transgenic, “floxed,” and epitope-tagged form of the gene using Cas9-mediated genome editing. To produce a repair plasmid, a recodonized version of bp 50 to 615 of the *GAP45* gene, containing a triple-HA tag within a predicted low-complexity region (46) and preceded by a SERA2lox71 intron (an adapted version of the SERA2-loxPint module described previously [52]), was synthesized commercially (GeneArt; Thermo). A synthetic *lox66* (Integrated DNA Technologies [IDT]) at the 3′ end and homology arms amplified from the 3′ and 5′ untranslated regions (UTRs) (and overlapping the 3′ and 5′ ends of the PfGAP45 [Pf stands for *P. falciparum*] coding sequence, respectively) were added to the recodonized *GAP45* by InFusion cloning (TaKaRa), and the resulting plasmid was linearized by restriction digest prior to transfection. The Cas9, single guide RNA (sgRNA), and selectable markers were introduced on a pDC2-based plasmid as described previously (53, 54). The GAACCTCTTGAACAAGAAC sequence toward the 5′ end of the endogenous *PfGAP45* gene, upstream of an AGG protospacer-adjacent motif, was used to target Cas9. For genetic complementation of the *GAP45:loxP* parasite clone, a cassette comprising the endogenous *PfGAP45* coding sequence and approximately 1 kb of the *PfGAP45* 5′ flanking sequence (presumed to contain the promoter sequence) was cloned upstream of the P. berghei dihydrofolate reductase (*PbDT*) 3′ UTR flanked by homology arms, targeting it to the *Pfs47* locus. This construct was used in conjunction with an sgRNA targeting the same locus, as described previously (54).

### P. falciparum culture and transfection.

All transgenic P. falciparum parasites used in this work were based on the 3D7 clone and express the DiCre recombinase components. The previously described 1G5DiCre parasite clone (45) was modified using a Cas9-based approach to remove approximately 1 kb of the displaced and duplicated 5′ end of the *SERA5* gene, in order to prevent spontaneous excision of the DiCre components driven by homologous recombination between the duplicated regions of *SERA5* gene sequences. The resulting cloned DiCre-expressing P. falciparum clone, called B11, was cultured in AlbuMAX-supplemented RPMI 1640 and synchronized according to standard procedures (55). Percoll-purified B11 schizonts were transfected with 15 µg pDC2 Cas9/gRNA/hDHFR (human dihydrofolate reductase)/yFCU (yeast cytosine deaminase/uridyl phosphoribosyl transferase)-containing plasmid and 30 µg linear repair template using the Amaxa 4-D electroporator and the P3 primary cell 4-D Nucleofector X kit (Lonza) as described previously (56). Twenty-four hours posttransfection, the culture medium was replaced with fresh medium containing WR99210 (2.5 nM), which was withdrawn after 4 days. The drug-selected line was cloned by limiting dilution using a plaque-based method (57). The *GAP45:loxP* clone was treated with Ancotil (1 µM), so as to remove any parasites still carrying the pDC2 yFCU-containing plasmid prior to transfection with the complementation construct used to generate the *GAP45:loxPcomp* clones.

### Flow cytometry.

For growth assays, parasitemia was determined using flow cytometry-based detection of Hoechst-stained DNA within RBCs. At the given time points, samples of the parasite culture were fixed with 4% paraformaldehyde (PFA)-0.02% glutaraldehyde for 1 h at room temperature. These samples were then stained with 4 µg/ml Hoechst solution for 30 min and analyzed on a BD Fortessa instrument. Erythrocytes were gated based on their forward and side scatter parameters, and Hoechst stain-positive RBCs were identified using the 450/50-UVA detector. For invasion assays, Percoll-purified schizonts were incubated with RBCs (~5% parasitemia) and incubated at 37°C in shaking cultures. Samples were fixed with 4% PFA-0.02% glutaraldehyde after 0, 2, and 24 h and stained with Sybr green I (Life Technologies) prior to analysis on a BD Fortessa instrument, using the 530/30-blue detector configuration.

### Western blotting.

For detecting schizont proteins, synchronized parasites were Percoll purified, washed, and lysed using saponin. Merozoites were then lysed with 0.1% SDS, 1% Triton X-100, 1× protease inhibitors in phosphate-buffered saline (PBS). Following centrifugation, the supernatants were mixed with sample buffer, incubated for 10 min at 95°C, and subjected to SDS-PAGE. Proteins were transferred to nitrocellulose membranes which were then blocked in 3% bovine serum albumin (BSA) in PBS containing 0.2% Tween 20 before staining with primary antibodies against GAP45, CDPK1, or the HA epitope (3F10; Roche). Membranes were washed and then incubated with the relevant horseradish peroxidase (HRP)-conjugated secondary antibodies and detected using a chemiluminescent reagent (Millipore).

Shedding of parasite proteins into culture supernatant was measured as a proxy for egress. Approximately 10^7^ schizonts per sample were incubated in 200 µl RPMI and allowed to undergo egress for 45 min. Parasite material was then pelleted by centrifugation, and the supernatant was passed through a 0.45-µm nylon filter (Costar). Supernatant samples were subjected to SDS-PAGE and transferred to nitrocellulose, and membranes were blocked as detailed above. A rabbit anti-SERA5 polyclonal antibody (58) was used in combination with an anti-rabbit-HRP conjugate to detect the released parasite proteins.

### IFA.

Schizonts were smeared onto glass slides and air dried, fixed with 4% paraformaldehyde for 30 min, permeabilized with 0.1% Triton X-100 in PBS for 10 min, and blocked overnight in 4% BSA in PBS prior to staining. Slides were incubated with combinations of the anti-HA monoclonal antibody (MAb) 3F10 (Roche), a rabbit anti-GAP45 serum, the human anti-MSP1 MAb X509 (59), a rabbit anti-GAP50 serum, rabbit anti-MTIP serum, or rat anti-MyoA serum; washed; and then incubated with appropriate Alexa-conjugated secondary antibodies (Life Technologies). Slides were mounted using ProLong Gold antifade mount with 4′,6-diamidino-2-phenylindole (DAPI; Life Technologies) and imaged using a Nikon Eclipse Ni microscope with a Hamamatsu C11440 digital camera. Images were processed using Fiji.

### Time-lapse video microscopy.

Egress was visualized by differential inference contrast (DIC) light microscopy as described previously (26) using a Nikon Eclipse Ni microscope with a Hamamatsu C11440 digital camera. The PKG (cGMP-dependent protein kinase G) inhibitor compound 2 (4-[7-[(dimethylamino)methyl]-2-(4-fluorphenyl)imidazo[1,2-α]pyridine-3-yl]pyrimidin-2-amine) was used to tightly synchronize parasites prior to egress, as described previously (26). To visualize egress in both DMSO- and RAP-treated cultures concurrently, one culture was stained with 1 µg/ml Hoechst stain (Thermo) for 5 min before washing and pooling the cultures and imaging, as previously described (30). The percentage of parasites undergoing egress and the time to egress were quantified from nine videos, and the statistical significance of any differences between the DMSO- and RAP-treated parasites was calculated using paired *t* tests.

### Transmission electron microscopy.

*GAP45-HA3:loxP* parasites were treated at ring stage with DMSO or RAP and allowed to develop to schizont stage. Schizonts were Percoll purified and incubated with 1 µM compound 2 for 4 h before fixation with 2.5% glutaraldehyde-4% formaldehyde in 0.1 M phosphate buffer (PB). Schizonts were embedded in 2% agarose and cut into 1-mm^3^ blocks, washed in PB, and transferred to a Biowave microwave for processing (Pelco; Agar Scientific). The schizont/agarose blocks were twice washed at 250 W for 40 s in PB, stained with 1% reduced osmium for 14 min under vacuum (with/without 100-W power at 2-min intervals), and then washed twice on the bench and twice in the microwave with PB. A further stain with 1% tannic acid for 14 min (with/without 100-W power at 2-min intervals under vacuum) was followed by a quench with 1% sodium sulfate at 250 W for 2 min under vacuum and bench and microwave washes in water (as for PB). The blocks were then dehydrated in a graded ethanol series (20%, 50%, 70%, 90%, and 100%, twice each) and then acetone (three times), at 250 W for 40 s under vacuum. Exchange into Epon resin (Taab Embed 812) was performed in steps of 25, 50, and 100% resin in acetone, at 250 W for 3 min, with vacuum cycling. Pure Epon was infiltrated in the three final microwave steps with the same settings, before a final overnight infiltration on a rotator and then baking for 24 h at 60°C. Sections (70 to 75 nm thick) were stained with lead citrate and imaged in a Tecnai Spirit BioTwin (FEI) transmission electron microscope (60).
